# Intraocular Implants for the Treatment of Autoimmune Uveitis

**DOI:** 10.3390/jfb6030650

**Published:** 2015-07-31

**Authors:** Darren J. Lee

**Affiliations:** Department of Ophthalmology/Dean McGee Eye Institute, University of Oklahoma Health Sciences Center, 608 Stanton L. Young Blvd, DMEI PA404, Oklahoma City, OK 73104, USA; E-Mail: Darren-lee@ouhsc.edu; Tel.: +1-405-271-3642; Fax: +1-405-271-8128

**Keywords:** uveitis, non-infectious uveitis, intraocular implant, uveitis treatment, ocular drug delivery device

## Abstract

Uveitis is the third leading cause of blindness in developed countries. Currently, the most widely used treatment of non-infectious uveitis is corticosteroids. Posterior uveitis and macular edema can be treated with intraocular injection of corticosteroids, however, this is problematic in chronic cases because of the need for repeat injections. Another option is systemic immunosuppressive therapies that have their own undesirable side effects. These systemic therapies result in a widespread suppression of the entire immune system, leaving the patient susceptible to infection. Therefore, an effective localized treatment option is preferred. With the recent advances in bioengineering, biodegradable polymers that allow for a slow sustained-release of a medication. These advances have culminated in drug delivery implants that are food and drug administration (FDA) approved for the treatment of non-infectious uveitis. In this review, we discuss the types of ocular implants available and some of the polymers used, implants used for the treatment of non-infectious uveitis, and bioengineered alternatives that are on the horizon.

## 1. Introduction

Uveitis is the third leading cause of blindness in developed countries [1,2,3], with an incidence of 52–93 cases per 100,000 persons per year and a prevalence of 115 cases per 100,000 persons [4,5]. Uveitis can affect different parts of the eye and the affected part distinguishes the different types of uveitis. Anterior uveitis involves the iris, cornea, and ciliary body, intermediate uveitis involves the vitreous and pars plana, and posterior uveitis is an inflammation of the retina [6,7]. Posterior uveitis can be devastating to vision and is difficult to diagnose and treat [8], whereas anterior uveitis is more common and can be easier to diagnose and treat [9]. Following an initial episode of anterior uveitis the recurrence rate is 36% for three or more episodes within five years [10]. Suppression of the inflammation can be achieved through the use of topical corticosteroids, but is an ineffective long-term solution because the steroids also cause cataracts and glaucoma [11,12,13]. The administration of systemic steroids is more effective than topical steroids for posterior uveitis and has a lower incidence of elevated intraocular pressure and cataracts [14]. However, systemic steroids can have much more serious side effects, such as weight gain, hyperglycemia, osteoporosis, gastrointestinal ulceration, intense mood changes, depression, and violent aggressive behavior [15].

The current treatment paradigm for autoimmune uveitis focuses on the use of non-steroidal immunosuppressive therapies to keep the ocular inflammation suppressed [1]. These therapies include anti-metabolites, such as methotrexate [2,16,17]; calcineurin inhibitors, such as cyclosporine A [2,18]; DNA alkylating agents, such as cyclophosphamide and chlorambucil [2,18]; and biologics, such as Adalimumab and Infliximab [19,20,21,22]. Most of these therapies are systemic treatments that are not specific for the eye so can cause systemic side effects. Therefore, a localized treatment approach has the advantage of avoiding systemic complications. Intravitreal injection of methotrexate or sirolimus is a localized treatment approach for chronic uveitis, but does not always lead to sustained suppression, so is not a viable long-term solution in every case [17,23,24]. Moreover, frequent invasive intravitreal injections can lead to retinal detachment, hemorrhage, or endophthalmitis. There is clearly a need for a localized treatment approach for autoimmune uveitis and retinal diseases. In this review, we will discuss the different types of FDA approved intraocular implants, some of the polymers involved in their composition, and what other implants are on the horizon.

## 2. Biodegradable Implants

An implantable long-lasting sustained release of medication is advantageous for treatment of ocular disease because it allows for effective delivery to the posterior region of the eye and eliminates the need for frequent intraocular injections of the medication. This sustained release can be achieved by imbedding a biodegradable polymer with the medication so as the polymer is degraded the medication is released. The process of converting a hydrophobic polymer into a water-soluble material is termed erosion. Erosion can occur through either surface-erosion or bulk-erosion. The type of erosion that occurs is dependent on the permeability of the polymer to water and the rate at which erosion of the polymer occurs. If the polymer erodes slower than water can penetrate into the core, the surface continues to erode as the molecular weight decreases and is termed surface-erosion [25]. In bulk-eroding polymers the water penetrates into the polymer faster than degradation of the polymer resulting in degradation of the entire material [25]. Because of the penetration of water into the polymer during bulk-erosion, interaction of the water with the medication can result in the destruction of the medication before it can be released. Another problem with bulk erosion polymers is auto-catalysis, in which the core is degraded quickly and once a pore is eroded the drug will be suddenly released—resulting in a sudden increase in drug release [25,26]. Since the type of polymers influences the surface or bulk erosion properties, the choice of materials is important to consider for the application. The bulk-erosion polymer would be useful for tissue engineering where a permeable membrane is useful for hydrolytic diffusion [27]. In the case of a desired sustained delivery, surface-eroding polymers would be more appropriate because of the stable drug release [28]. Biodegradable implants are advantageous because they do not require removal when the drug has been exhausted [25].

### 2.1. Bulk-Eroding Polymers

Polyglycolide or poly(glycolic acid) (PGA) has been used as a degradable suture since 1970 [29]. Glycolic acid is readily incorporated by cells through the citric acid cycle [30]. However, it can affect the pH and elicit an inflammatory response [31,32,33], so can be problematic for ocular implants.

Polylactide or poly(lactic acid) (PLA) has four different chiral forms [34]. Depending on the chirality, PLA can take 1–5 years or more to completely degrade [35]. Because of the extended degradation time research on PLA has been limited as such, PLA alone has not been explored as a potential drug delivery system [36,37,38,39]. Instead, PLA can be combined with PGA to form poly(lactide-co-glycolide) (PLGA) in order to accelerate the degradation time. Moreover, by changing the ratio of PGA to PLA the degradation time can be adjusted from 1–2 months to 5–6 months [40]. PLGA has been used for sutures since 1974 [41], and has been used for the delivery of proteins [42,43,44,45], anti-inflammatory drugs [46,47], and siRNA [48,49,50]. Unfortunately, two drawbacks to PLGA is that PLGA degradation products can both lower the pH and bulk-erosion can result in a sudden increase of the drug and destruction of the medication due to water that has diffused into the matrix [51,52,53].

Polycarpolactones (PCL) have been used as a contraceptive that delivers levonorgestrel for over a year and has been on the market for over 25 years [54]. Because degradation of micro-particles and nano-particles occurs over 2–3 years, blending with other polymers, such as PLGA is done to accelerate erosion [49,55,56].

### 2.2. Surface-Eroding Polymers

Polyanhydrides are a class of polymers that contain two carbonyl groups joined by an ether bond [34]. Hydrolysis of this bond results in two carboxylic acids that are readily metabolized, so can lower the pH. Polyanhydrides are unique because the polymer backbone has a direct relationship with the degradation rate that can vary by more than six orders of magnitude [56,57]. Combining aromatic and aliphatic diacids can slow degradation and extend drug delivery from days to years [28].

Polyacetals have two ether bonds connected to the same carbon (germinal) and maintain a milder pH because they do not degrade into carboxylic acids [58]. Some degrade when entering the lysosome at pH 4–5 [58] and have been used to deliver siRNA, DNA, and proteins for acute inflammation [59,60,61,62,63,64,65,66]. Despite these properties, polyacetals have found limited used because they do not meet the mechanical strength needs of most implant applications.

Polycarbonates are very stable and consist of two geminal ether bonds and a carbonyl bond. It is thought that enzymatic degradation is responsible for surface erosion of this polymer [67]. Poly(trimethylene carbonate) (PTMC) is the most characterized polycarbonate [68]. In order to increase the mechanical and degradation properties, PTMC is copolymerized with PLA, PCL, polyether, or poly(L-glutamic acid) [69,70,71,72,73,74]. The copolymer used can give the polycarbonate drastically different mechanical properties. A gel that rapidly degrades can be made with polycarbonate and dihydroxacetone for use in clotting [75]. In contrast, significant mechanical strength and slow degradation can be achieved if the copolymer is a tyrosine-derivative, this can be used in tissue engineering of bone or muscle [76,77,78,79,80,81,82,83,84].

## 3. Non-Biodegradable Implants

The use of biodegradable implants for ocular disease has been limited because intravitreal injection of PLGA microspheres can cloud vision [85] and movement of the implant into the anterior chamber or in front of the retina can be a complication [8,86]. Some non-biodegradable implants can be anchored to the sclera for easy removal and to prevent the implant from mobilizing into an inconvenient position [87]. The variable drug release kinetics associated with biodegradable polymers [8,26] can be avoided by coating the polymer with a non-biodegradable polymer or through storage of the drug in a reservoir encased in a non-biodegradable polymer [8]. The coating can be porous or have a small hole to allow for a small area of diffusion, these typically have an initial burst that is followed by a consistent release of the medication [8,25,88,89]. A depleted non-biodegradable implant has a risk of irritating the tissue or eliciting an inflammatory response, so requires a second procedure to have the implant removed [25,90].

Silicon and ethylene-vinyl acetate copolymer (EVA) are used as hydrophobic membranes for non-biodegradable implants [8,91,92]. Poly(vinyl alcohol) (PVA) is more hydrophilic, so is more permeable to a wider range of drugs [88]. Polyimide has been used for a variety of applications from photovoltaic cells to biomedical implants [8,25,93]. Poly(methyl methacrylate) (PMMA) is a clear plastic that can be used for drug delivery [94]. Phosphatidylglycerol is a negatively charged phospholipid that can be used as a vehicle for drug delivery [95].

## 4. FDA Approved Implants for Uveitis

Ozurdex^®^ is sold by Allergan and has been FDA approved to treat macular edema secondary to branch or central retinal vein occlusion and non-infectious posterior uveitis. Dexamethosone is released from a PLGA matrix for up to four months (Table 1) and because it is completely biodegradable it does not need to be removed at a later date [87]. No patients that received the Ozurdex^®^ implant required intra-ocular pressure (IOP) lowering medications or surgery, and only 17% of patients experienced an increase in IOP of 10 mmHg or more [96,97,98]. Migration of the Ozurdex^®^ implant to the anterior chamber has been reported [86,99]. However, it is possible that this complication can be avoided with careful screening of patients for post-lensectomy-vitrectomy aphakia [100,101].

Retisert^®^ has been developed by pSivdea Corp. (Watertown, MA, USA) and is a non-biodegradable implant that delivers fluocinolone acetonide. It has been FDA approved for noninfectious posterior uveitis. The PVA and silicon coating allows for consistent release of fluocinolone for up to 30 months (Table 1) [87,101]. The Retisert^®^ implant has been associated with the development of cataracts [102] and an increase in IOP of more that 10 mmHg in more than 60% of patients [102,103,104].

**Table 1:** FDA approved intraocular implants.

Implant	Medication	Method of Implantation	Size	Release Time	Reference
Ozurdex	Dexamethosone	22 gauge designer applicator	rod-shaped, 0.46 mm diameter, 6 mm long	up to 6 months	[25,101]
Retisert	Fluocinolone acetonide	surgical insertion	1.5 mm diameter, 6 mm long, 2 mm wide	up to 2.5 years	[25,30,101,104]
IIuvien	Fluocinolone acetonide	injection with 25 gauge needle	3.5 mm × 0.37 mm tube	up to 3 years	[8,25,87,101]
Vitrasert	Gancyclovir	surgical insertion	4–5 sclerotomy	up to 8 months	[25,101,105]
Surodex *	Dexamethosone	25 gauge needle, placed during cataract surgery	1 mm × 0.5 mm	7–10 days	[25,101]

* Surodex has been approved for use in China and Singapore.

## 5. FDA Approved Implants for Ocular Disease

Iluvien™ is a non-biodegradable implant that delivers fluocinolone acetate that is in a PVA matrix within a polyimide tube. The tube has membrane caps on each end to allow for diffusion of water into the matrix. Iluvien has been FDA approved for diabetic macular edema, and delivers medication for up to 36 months (Table 1) [8,87,101].

Vitrasert^®^ contains gancyclovir in a PVA matrix with a non-biodegradable EVA coating. Gancyclovir is delivered for 5–8 months (Table 1) and is effective in the treatment of CMV [101,105,106].

Surodex^®^ is a poly(lactic-glycolic acid) device approved for use in China and Singapore that is used to control post-cataract surgery inflammation. This is inserted in the posterior or anterior chamber during cataract surgery and dexamethosone is delivered for up to 10 days (Table 1) [101,107,108,109].

## 6. Implants in Development

While the above-discussed implants have been effective for the treatment of uveitis and other ocular diseases, these devices still have additional challenges. For instance, if elevated intraocular pressure control is a concern, these corticosteroid delivery devices should be carefully considered [97,100,102,103,104]. In this case topical steroid use is preferred over injectable steroids because the topical steroids can be discontinued if an increase in pressure occurs. Therefore, a tunable implant that allows for control over the delivery of medication is advantageous to avoid unnecessary delivery of the drug. This type of device would also be advantageous because uveitis is a relapsing and remitting disease and it would be attractive to be able to control the drug concentration depending on the disease state [110]. We also discuss the development of non-steroidal immunosuppressive drug delivery devices.

### 6.1. Non-Steroid Implants

In order to avoid the side effects associated with sustained corticosteroid use it would be advantageous to have a non-steroidal implant to deliver a localized immunosuppressant to the eye. Methotrexate has been used safely and effectively as a systemic treatment for noninfectious uveitis for years [16]. It can also be injected into the vitreous as a localized treatment for uveitis [17,23,24]. A nanogel of PEGylated poly ethyleneimine containing methotrexate has shown to be effective in reducing joint inflammation in a murine model of arthritis [111]. Cyclosporine A is a calcineurin inhibitor that has been used as a systemic treatment for uveitis [2,18]. PCL and PGLC nanoparticles are being developed as a vehicle to deliver cyclosporine A as an injection into the subconjunctiva or vitreous [112,113]. There has been some investigation into tethering neutralizing antibodies such as, anti-TNF, to polymers [112,114]. If these non-steroidal immunosuppressive medications could be adapted as an ocular implant they would provide an excellent alternative to the current sustained corticosteroid delivery devices.

Another interesting device in development is NT-501, through Neurotech, Inc. NT-501 contains polyethylene terephthalate yarn that is loaded with retinal pigmented epithelial cells (RPE). The polyethylene terephthalate yarn is contained within a polysulfone [8,88] membrane that is sutured with a titanium loop to the scleral wall. The semipermeable membrane allows for the diffusion of nutrients into the device to sustain the RPE cells and diffusion of RPE products out into the vitreous. Neurotech is developing this device for the treatment of retinitis pigmentosa, so the RPE is genetically engineered to produce recombinant CTNF or VEGF neutralizing antibodies or both [25,115,116,117]. If the RPE cells could be genetically engineered to secrete steroidal or non-steroidal immunosuppressive drugs, this could be another excellent implant for the treatment of uveitis.

### 6.2. Tunable Implants

A healthy eye has a clear cornea and lens that allows for the passage of light to the retina [118]. This property can be exploited to deliver a specific wavelength of light to an implant in the vitreous. Polymers can be formulated to include light sensitive components that allow for a permanent or temporary change in the chemistry or structure of the polymer matrix to either trigger drug release or prevent drug release [8,119]. Temporary photo-activated changes are achieved with chromophores that allow for drug release only in the presence of the photo-stimulus because when that specific wavelength of light is removed the chromophore returns to the stable state [8,120]. In contrast, an irreversible change occurs with pyrene derivatives when the light is removed [120]. Another type of photo tunable system is to convert the light into thermal energy. This can be achieved by coating the polymer with a nontransparent metal that converts the light into thermal energy [121]. The thermal energy then breaks down the polymer to create permeable pores or an orifice for the drug to diffuse out. This photo-activated technology is limited because wavelengths of light more than 900 nm cannot penetrate the eye, and wavelengths too short can cause damage to ocular structures [122]. In addition, many of the chromophores are too toxic to use in biological systems [8].

Another noninvasive method to achieve precise control of drug release from a polymer matrix is with magnetic fields. Magnetically modulated systems for drug release utilize a matrix or reservoir-based design. The matrix systems consist of magnetic particles imbedded in the polymer matrix. Upon exposure to a magnetic field, the magnetic particles vibrate in the pores to increase the pore size and allow for a greater rate of drug release [123]. The reservoir-based device contains one or more magnetic components that allows for modulating the diffusion of the drug with an external magnetic field [124]. Repeated usage of these devices results in a reduced magnetic response [125], so long term usage is not practical. Moreover, these devices would be problematic if computerized tomography (CT) and magnetic resonance imaging (MRI). There is potential for this technology, but further research is necessary before it can be implemented in the clinic.

Conductive polymers have both polymer and metal properties [8,126]. Since 1977, conducting polymers have been studied for many biomedical applications, in particular for the electrically tunable property for drug delivery [127,128]. Electric stimulation alters the redox state of the polymer to affect the charge, volume, permeability and hydrophobicity. Because the volume of the polymer can be altered it is possible to contain the drug in a reservoir and upon electrical stimulation the volume change causes the drug to be released from the polymer [129]. These polymers are biocompatible, non-toxic, and allow for fine control of drug release [130]. They are also non-biodegradable and can be altered to biodegrade, but at the expense of lowering the conductivity, and drug release capacity [131]. The disadvantage of conductive polymers is that the power source requires a bulky battery and wires to actuate the device.

The Micro Electro Mechanical System (MEMS) is an implant that achieves actuation through temperature, electrical stimulus, magnetic field, or osmotic pressure [124,132,133,134]. These devices consist of a reservoir and an actuator to mechanically push the medication out of the reservoir upon proper stimulation. Ideally, these devices have the advantage of being able to refill the reservoir [8] and have lower power requirements, so a wireless signal could be employed [124,135]. Unfortunately, MEMS are still in the developmental stages as the reservoir is small and the lifetime is less than a year [25]. However, a new model is in clinical trials that could last up to five years [25]. There is also a magnetic stimuli responsive MEMS implant being investigated to deliver medication to the posterior of the eye [124]. However, the long-term feasibility of this magnetic device is still to be determined as discussed with the magnetically modulated systems.

## 7. Summary

The current treatment paradigm for chronic noninfectious uveitis is to suppress the inflammation with localized or systemic immunosuppressive medications for a period of time that is sufficient for the patient to be slowly weaned off of the medications [1]. Presumably, during the time that the uveitis is suppressed the patient establishes a regulatory immune response to provide a resistance to relapse [136,137,138,139]. It is also probable that some aspects of ocular immune privilege are re-established during this period of immunosuppression [140].

If medication is providing systemic immunosuppression the patient will require careful monitoring to ensure systemic side effects do not occur [15]. Systemic side effects may be severe enough that termination of a treatment may be necessary and will often be related to a relapse. This is where localized treatments are ideally suited for uveitis patients. We have discussed several ocular implants for the treatment of uveitis and other ocular disease. Another advantage of ocular implants is that they are effective in delivering drugs to the retina [8].

The implants available for the treatment of ocular inflammation are either biodegradable or non-biodegradable. Biodegradable implants have the advantage of only requiring one procedure to install the implant, the disadvantage is that the implant can move and the release rate of the medication is not consistent [8,25]. The non-biodegradable implants allow for a continuous release rate and some can be secured to the sclera to prevent movement away from the implant site and for ease of removal [8,25,87]. Both types of implants have their advantages and disadvantages so until additional advancement can eliminate the disadvantages the ophthalmologist will need to evaluate the implants carefully before choosing which is more appropriate for the particular patient.

Current ocular implants available for the treatment of ocular inflammation deliver dexamethasone or fluocinolone acetate, both of which can trigger an increase in IOP [96,97,100,102,103,104,141]. This can be problematic, especially if a patient has steroid induced glaucoma. Fortunately, there are additional implants in development that deliver non-steroidal medication as an alternative to the current implants available. Polymers imbedded with non-steroidal immunosuppressive drugs, such as cyclosporine A and anti-TNF are in development and could provide an excellent alternative to the current sustained release corticosteroid delivery devices available for the treatment of uveitis and other ocular disease [88]. The development of tunable ocular implants is desirable because the ability to control the drug release can help to reduce side effects and can extend the availability of drug in the implant, thereby extending the life of the device [8]. Another interesting device is NT-501, currently in clinical testing by Neurotech, Inc. NT-501 is in development for retinitis pigmentosa, but if found to be a feasible long-term treatment, it could be adapted to function for ocular inflammatory disease as well.

The availability of implantable corticosteroid delivery devices has improved the outcome for noninfectious uveitis patients, particularly those with posterior uveitis [100,141]. This represents an exciting area of research and the success of the current devices available has bolstered an interest in the development of additional implants for the treatment of uveitis and other ocular diseases. In the next 4–5 years, we should see the translation of many new drug delivery devices into early stage implantable systems and we hope that many more ocular implants that are currently in clinical trials will become available for the treatment of ocular inflammatory diseases.
